# Severe ME in Children

**DOI:** 10.3390/healthcare8030211

**Published:** 2020-07-14

**Authors:** Nigel Speight

**Affiliations:** Paediatrician, Southlands, Gilesgate, Durham DH1 1QN, UK; speight@doctors.org.uk

**Keywords:** Myalgic Encephalomyelitis (ME), Chronic Fatigue Syndrome (CFS), Severe ME, Very Severe ME, Post-Exertional Malaise (PEM), Fabricated and Induced Illness, Cognitive Behavioural Therapy, Graded Exercise Therapy, Pervasive Refusal Syndrome, immunoglobulin

## Abstract

A current problem regarding Myalgic Encephalomyelitis/Chronic Fatigue Syndrome (ME/CFS) is the large proportion of doctors that are either not trained or refuse to recognize ME/CFS as a genuine clinical entity, and as a result do not diagnose it. An additional problem is that most of the clinical and research studies currently available on ME are focused on patients who are ambulant and able to attend clinics and there is very limited data on patients who are very severe (housebound or bedbound), despite the fact that they constitute an estimated 25% of all ME/CFS cases. This author has personal experience of managing and advising on numerous cases of severe paediatric ME, and offers a series of case reports of individual cases as a means of illustrating various points regarding clinical presentation, together with general principles of appropriate management.

## 1. Definitions

Empirically, one can regard Severe Myalgic Encephalomyelitis/Chronic Fatigue Syndrome (ME/CFS) as affecting a patient who is housebound and bedbound most of the time, functioning at approximately 5–15% of normal capacity.

There are also even less fortunate patients who are totally bedbound, suffering continuous extremely unpleasant symptoms and in need of nursing care to meet all their needs for nutrition and personal hygiene. This can be termed Very Severe ME, and patients’ functional capacity is less than 5%.

Paradoxically, the cardinal diagnostic feature of ME/CFS, Post-Exertional Malaise (PEM), is difficult to elicit in the very severely affected, as the patient is hardly capable of the slightest exertion. However, in common with all ME sufferers, there is usually evidence of deterioration after even minor forms of stress, such as light, sound, odour or motion. Hypersensitivities to various types of stimuli can cause “sensory overload” and can result in a “crash”—a period of immobilising mental or physical debility—even in patients who are not normally very severely affected [1].

Those in the group that are “only” severely affected *do* experience PEM to a marked degree, often in terms of increased intensity of pain.

## 2. Problems and Challenges for the Patient with Severe ME/CFS

Patients with ME have often suffered from disbelief on the part of their medical attendants, and even those severely affected are not protected from this disbelief.

This disbelief can operate at several different levels.

The first level is when the doctor disbelieves that ME/CFS even exists as an organic illness in the first place. This can lead to inappropriate referral to psychiatrists, and then to harmful management plans.

The next level is for the doctor to accept that ME/CFS exists, but to disbelieve that a particular patient has it.

A further level is to have an overoptimistic view on the efficacy of certain forms of “treatment”, usually Cognitive Behavioural Therapy (CBT) and/or Graded Exercise Therapy (GET). This leads to the patient having the diagnosis of ME/CFS withdrawn when they fail to respond to this management strategy, or as so often happens when the patient’s condition worsens. GET is especially likely to lead to such deterioration. Rather than drawing the conclusion that these “therapies” are misguided, the doctor often changes the diagnosis to one involving a psychological causation.

The final level is when the doctor diagnoses ME/CFS at a stage when it is still mild or moderate, and then the patient’s condition worsens to the extent that it becomes severe. The doctor can then lose confidence in the original diagnosis, and change this to a psychological diagnosis such as Pervasive Refusal Syndrome (as in Case A below).

For the patient with Severe ME/CFS to be subjected to such disbelief is of course adding insult to injury.

The worst scenario is when the paediatrician, as a result of some of the above varieties of disbelief, makes a provisional diagnosis of Fabricated and Induced Illness (FII), and refers the patient to Social Services as a case needing safeguarding. This can lead to threats to remove the young person from their family. This is currently increasingly fashionable in the UK [2].

## 3. Challenges for the Doctor Faced with a Case of Severe ME/CFS

If the doctor has never seen a case of Severe ME/CFS before, as is often the case, there is a natural tendency to panic, and to worry that the patient might die, or that the diagnosis was wrong. In addition, the doctor may feel a sense of helplessness, with no obvious therapeutic options available. These emotions can lead to the doctor adopting a strategy of avoidance, refusing to accept that he/she has a duty of care to the patient, and regrettably many patients with Severe ME/CFS suffer near total neglect by their doctors. Alternatively, the doctor may try to insist on inappropriate “treatment” strategies, such as CBT or GET. Neither form of treatment has been studied in the severe or very severe population. Further, especially in the severe cases, rigidly enforced GET is nearly always seriously harmful. The current NICE Guidelines (2007) specifically do *not* recommend GET in Severe ME [3].

## 4. Possible Clinical Features of Severe and Very Severe ME/CFS

- Minimal energy levels, resulting in the patient being housebound or bedridden;
- Paralysis;
- Severe generalised continuous pain;
- Severe continuous headache;
- Hyperaesthesia/extreme sensitivity to touch;
- Abdominal pain, worse after food—this may be so severe as to interfere with nutrition;
- Sleep disturbance, possible hypersomnolence or difficulty sleeping on account of pain and headache;
- Major problems with cognition, concentration and short-term memory;
- Extreme sensitivity to light and sound;
- Multiple chemical sensitivities;
- Problems with eating and drinking—this can be due to either general weakness or actual dysphagia, and this may necessitate tube feeding;
- Aphonia (mechanism unclear);
- Myoclonic jerks;
- Incontinence.

## 5. General Points Regarding Impact of Symptoms on Quality of Life

It has been estimated that the sheer severity of suffering experienced by patients with the above symptoms can actually be worse than that suffered by patients with other chronic conditions such as multiple sclerosis and cancer [4].

The severity of the photosensitivity can be a further trigger to disbelief, as the doctor may find it difficult to accept that the patient not only has to lie in a darkened room but has to wear eye protection in addition.

The abdominal pain may be so severe as to interfere with nutrition, and some cases are due to an added complication, Mast Cell Activation Disorder [5]. This has probably been responsible for some actual fatalities. Specific treatment for MCAS includes oral cromoglycate and antihistamines.

Of course, simply having ME/CFS does not protect one from other causes of post-prandial abdominal pain, such as median arcuate ligament syndrome (MALS) [6], or Helicobacter pylori gastritis.

## 6. My First Severe Case

I will describe this in some detail as I made every mistake in the book.

When first seen, this 13-year-old girl was only moderately severe, being well enough to attend my clinic. A few months later she suffered marked deterioration, probably due to a virus infection. She was bedridden and suffered many of the symptoms listed above.

She had markedly raised antibodies to coxsackie B (1:520), which remained elevated at that level for over a year.

My first mistake was to panic, and to think that I might have been mistaken in my initial diagnosis. I accordingly asked an immunologist colleague in our regional hospital for a second opinion, as he had previously confirmed to me that he “believed” in ME/CFS. The girl and her parents were quite dubious about the referral as it involved a 20 mile journey, and they knew instinctively that this would be unpleasant for her and might well make her worse.

I got my colleague to promise that she would be seen the same day and then allowed straight home.

The immunologist made the same mistake as I had and asked for second and third opinions, with the result that she was kept in the regional hospital for three days, had numerous investigations, and was seen and examined by numerous doctors. One neurologist insisted on her having to get out of bed and walk for him. (One year later, aged 14, she wrote him a dignified letter of reproach, to which he had the grace to respond.)

No new diagnosis was proffered and she was allowed home, the entire exercise having been a negative one for her. The whole experience was so traumatic for her that it took her 2–3 years to forgive both me and her parents properly.

I continued to make similar mistakes, by asking in an experienced GP with a special interest in ME/CFS. I knew him as a fine and kind doctor but in the event, he upset her further by insisting on looking into her fundi and then talking about a similar patient he once had who had died.

Next to upset her was the district nurse, who bustled in, said she had read all about ME and all would be fine if she made an effort to walk every day,

The family doctor then convened a meeting, at which the Health Visitor (children’s nurse) wondered whether it could all be due to father sexually abusing his daughter. This was duly considered, recorded in the minutes, and the minutes sent to the family, to their predictable outrage.

The main lesson I learnt from all this was how many ways it is possible to upset a severely ill ME patient, and that one needs to be proactively protective of one’s patient, especially against other professionals, and disbelieving friends and relatives.

For professionals affected by “Furor Therapeuticus”, i.e., the feeling that one has to do something, it is better to remember that there is no proven curative treatment, and that they should perhaps concentrate on the “Do No Harm” element in the Hippocratic Oath.

In this case, I gradually regained my patient’s trust, with the help of a new family doctor. He and I shared the home visiting duties, going in alternately every 4 weeks or so.

Over the next six months, she continued to deteriorate and she found it increasingly difficult to eat and drink.

My next mistake was to give in for too long to her objections to nasogastric tube feeding. Once I finally persuaded her, life became much better for everyone.

Because of the evidence that immunoglobulin might be an effective therapy, she was treated with this by intramuscular injection, monthly, for 12 months. She made a very slow recovery, and tube feeding was stopped after 3 years. She continued to recover and 20 years later has a full-time job, and is operating at 95% of normal.

The final lesson to learn from this case is that virtually full recovery is possible even in severe cases, and one can use this fact to maintain hope for other similarly affected patients.

## 7. My Second Severe Case

This case is noteworthy as it demonstrates how much better things can go when the above lessons have been learnt. Again, the patient was a 13-year-old girl. Her presentation was unusual in that she presented acutely with severe loin pain, bad enough to be admitted with a presumptive diagnosis of renal colic. This diagnosis was excluded (I now think this illness started with a form of Bornholm Disease—convalescent titres against enterovirus were markedly elevated).

It soon transpired that she had multiple additional severe symptoms, including total prostration together with photophobia, generalised pain and hyperaesthesia. She was therefore given a presumptive diagnosis of ME/CFS. She was discharged home as soon as possible and, for the next year, needed round-the-clock nursing from her mother, who was herself a nurse. Tube feeding was started early to good effect, amitriptyline and carbamazepine were tried for her pain, and immunoglobulin was given.

After 12 months, she began to improve, and then her rate of improvement accelerated. By the end of two years, she had made a full recovery, and has never subsequently relapsed.

## 8. My Most Severe Case

Five years later, another 13-year-old girl presented. She was extremely ill, and I felt I had to get her seen by a colleague for a second opinion, and have a quick cranial MRI. Both occurred without the need for hospital admission. Over subsequent weeks, she lay motionless and in severe pain, and her breathing was so shallow that I was afraid she was going to die. Tube feeding and immunoglobulin were resorted to early on. In addition, I added clarithromycin, because of the work of Garth Nicolson from the US [7], in which he showed some cases of ME/CFS are due to atypical organisms including Mycoplasmas. She remained in this state for the next 9 months, with her mother providing total nursing care.

At nine months, I changed the clarithromycin to doxycycline, on the grounds that it was just possible that she might have a form of Lyme disease. From that time, she steadily improved, and within 12 months, she had recovered completely. At follow up 12 years later, she had suffered no relapses and was the healthy mother of a healthy child.

I am not sure one should draw too many conclusions from this case. However, it does suggest that total therapeutic nihilism is not the only approach, and that if one can think of therapeutic options that are probably harmless, one has a duty to consider them.

## 9. Overview and Discussion of These Cases

It is perhaps fortuitous that all these cases made good recoveries, and this may be a testament to the efficacy of immunoglobulin, for which further studies would seem to be indicated. However, many cases are not so fortunate, and remain severely or very severely affected for many years.

It is perhaps important to emphasise the importance of early diagnosis so that correct advice can be given from the outset. In other words, it is important to ignore those definitions which demand a duration of 6 months of symptoms before the patient can be given the diagnosis. Like so many “gold standard” definitions, these are more designed for those performing research rather than day-to-day clinical practice.

The correct advice is to rest in the early acute stage of the disease, rather than to attempt to fight one’s way out of it.

Another factor that can lead to delays in diagnosis is the felt need on the part of the doctor to exclude a large number of other conditions. It is perhaps better to make an early *provisional* diagnosis of ME on the balance of strong probability, while keeping an open mind for the future. ME should be regarded as a positive clinical diagnosis based on a careful history rather than a diagnosis of exclusion.

## 10. The Value of Home Visiting by the Doctor

The magnitude of the challenge posed by a case of Severe ME can produce an avoidance reaction on the part of some doctors. Many patients are virtually abandoned by both their family doctors and local and regional specialists.

In the UK, the practice of doing home visits seems to have gone into decline, both with general practitioners and hospital consultants. This would seem to be regrettable, especially in the case of Severe ME. The above cases all seemed to benefit from the ongoing home visits they received, both for moral support and symptomatic treatment. Sadly, many cases of Severe ME lie at home without having seen a doctor for many years.

## 11. Symptomatic and Supportive Treatment

This is an area where the art of medicine comes into its own. Individual variation in response to treatment is common, and the best approach is a policy of a cautious therapeutic trial. Symptoms that deserve attempts to mitigate include:

- Pain—this is thought to be of neuropathic type, and drugs that can be tried include anticonvulsants, and tricyclic antidepressants such as amitriptyline (which can also help with sleep). Very severe cases deserve opiates.
- Sleep—here it is justified to use hypnotics and/or melatonin to try to increase the duration and quality of sleep, and reduce the wakeful hours of pain.
- Headache—here it is important to see whether some of the headache is migrainous in origin, in which case prophylactic drugs and or dietary change may help.
- Intercurrent illnesses—ME/CFS does not protect from these, and a supportive family doctor willing to do home visits is worth his/her weight in gold. As the patient is too unwell to attend the GP surgery, this is the only way episodes of intercurrent infections such as tonsillitis and otitis media will be treated.
- Depression—naturally, severely affected ME patients can suffer from depression as a *secondary* result of their condition (I am surprised how few actually do). Cautious use of antidepressants in selected cases may be worthwhile.

## 12. Caution Regarding Dosage

Most ME patients appear to be abnormally sensitive to a wide range of drugs, developing side effects at quite low doses. Accordingly, it is wise to start at very low doses and titrate upwards with all due caution.

Further guidance on lists of drugs and appropriate doses can be found in the Paediatric Primer on ME/CFS in children and young people [8].

## 13. The Role of Physiotherapy

Having already stressed the real dangers of active physiotherapy/GET, it must be stated that there is a role for gentle physiotherapy and massage, involving passive movements to reduce the risk of joint contractures and venous thrombosis.

## 14. The Risk of Osteoporosis

These patients are at risk of osteoporosis because of being bedbound for prolonged periods. Consideration should be given to use of bisphosphonates, without insisting on an attendance at hospital for a bone density scan.

## 15. Vitamin D Deficiency

Again prolonged bedrest in a darkened room puts Severe ME/CFS patients at risk of Vitamin D deficiency, which should accordingly be anticipated and prevented.

## 16. How Not to Manage Severe ME/CFS

It is sometimes true that one can learn more from a bad example than a good one. Here are presented a sequence of cases where the management was less than ideal.

### 16.1. Case A

Yet again, this case involves a 13-year-old girl. Like the first case, she initially presented as only moderately severe. The paediatrician diagnosed her as ME and was managing her appropriately. Her condition then deteriorated markedly and the paediatrician panicked. Unfortunately, the form her panic took was to refer her to the local Child Psychiatrist. This latter took an extremely dogmatic position, and promptly dismissed the original diagnosis of ME/CFS, replacing it with the alternative psychiatric diagnosis of Pervasive Refusal Syndrome.

The psychiatrist then took out an emergency court order to enforce admission to her psychiatric unit against the parents’ and the patient’s wishes. When visited a few days later, the patient presented a truly pitiable sight. She was already being tube fed. She was extremely sensitive to sound and was using ear defenders. However, the psychiatrist banned these on the grounds that she needed to be “desensitised” against this problem. The same approach was applied to her light sensitivity, so the nurses were told not to allow her to use eye shades. She was being nursed on the open ward close to the main ward door which kept opening and shutting. Every time it shut, the noise caused a convulsive myoclonic jerk which affected her whole body.

The diagnosis of Pervasive Refusal Syndrome was refuted by Dr Bryan Lask, the Child Psychiatrist who had first described the syndrome. He pointed out that as she was accepting tube feeding, she could hardly be said to have *pervasive* refusal. The court order was dropped and she was transferred to a private nursing home. Here, she received Tender Loving Care (TLC), and made a full spontaneous recovery.

### 16.2. Case B

A 16-year-old girl already had Severe ME when a new paediatrician took over her care. She (the paediatrician) could not believe that anyone could be so light sensitive as to have to lie in a darkened room and still wear eye shades. She applied to the court to get the girl admitted to her hospital ward for active physiotherapy, and assured the judge that with this treatment she would be back at school full time within 6 months. The judge granted the order and the girl was admitted to hospital. She received active physiotherapy for three months and her condition deteriorated even further. Before every session, she told the physiotherapist that she was in breach of her professional ethical guidelines.

The judge eventually lifted the court order and the girl was allowed home, in a significantly worse state than when she was admitted. Twenty years later, she remains severely affected, still being bedridden and needing tube feeding.

This case shows a combination of disbelief in the reality of Severe ME combined with a false belief in the efficacy of Graded Exercise Therapy.

### 16.3. Case C

This 29-year-old young woman had had Severe ME since childhood. For all this time, she had received excellent empathetic care from her family doctor and her physician. She was in severe pain requiring opiates, and was nursed on a ripple bed. She had a urinary catheter in place and received nutrition via a nasojejunal tube.

Despite the severity of her condition, with fierce intelligence and determination, she managed to write a book for doctors and families on how Severe ME should be managed [9].

I remember meeting her around this time and thinking that it only needed one extra stressor to tip her over the brink and cause her death.

Sadly, a few months later, she developed renal colic, which necessitated her admission to hospital. Her own physician was not allowed to manage her (for some reason of internal hospital politics). The physician assigned to her care freely admitted he knew nothing about ME/CFS, and although she was nursed in a side room, she was still subjected to sensory overload. The physician used to come to her bedside with his entourage and engage in long arguments with her. She died of her ME in hospital a few weeks later.

This case demonstrates that it is not only emotional upset that can result from maltreatment, but very real medical harm to an extent that can be life threatening.

## 17. Take Home Messages

- Severe ME constitutes a major challenge for both patient and doctor.
- Mismanagement in the form of “activation regimes” can result in permanent harm or even death of the patient.
- The patient deserves the total commitment of one doctor, who is willing to visit at home on a regular basis.
- Referral to a psychiatrist who does not believe in ME/CFS can be harmful.
- The patient should be protected from sensory overload.
- The doctor should resist the temptation to overinvestigate, or involve too many other professionals.
- Nursing at home is usually far preferable to admission to a busy general hospital.
- Tube feeding is indicated when the patient has problems with eating and drinking.
- Urinary catherization may be helpful in reducing the stress of having to micturate.
- Symptomatic treatment for pain and sleep problems is worthwhile.
- Full recovery is possible.
- The role of immunoglobulin deserves further study [10].
- There is a need to improve both undergraduate and postgraduate medical training in this area, and to provide greater resources for the patient population affected.

## References

[1] Carruthers B.M., Jain A.K., De Meirleir K.L., Peterson D.L., Klimas N.G., Lerner A.M., Bested A.C., Flor-Henry P., Joshi P., Powles A.C.P. (2003). Myalgic Encephalomyelitis/Chronic Fatigue Syndrome; Clinical Working Case Definitions, Diagnostic and Treatment Protocols. J. Chronic Fatigue Syndr..

[2] Speight N. (2020). Myalgic Encephalomyelitis in the young—Time to repent. Acta Paediatr..

[3] Chronic Fatigue Syndrome/Myalgic Encephalomyelitis (or Encephalopathy): Diagnosis and Management. www.nice.org.uk/guidance/cg53.

[4] Nacul L.C., Lacerda E.M., Campion P., Pheby D., de L Drachler M., Leite J.C., Poland F., Howe A., Fayyaz S., Molokhia M. (2011). The functional status and wellbeing of people with myalgic encephalomyelitis/chronic fatigue syndrome and their carers. BMC Public Health.

[5] Weinstock L.B., Pace L.A., Rezaie A., Afrin L.B., Molderings G.J. (2020). Mast Cell Activation Syndrome: A Primer for the Gastroenterologist. Dig. Dis. Sci..

[6] Mak G.Z., Speaker C., Anderson K., Stiles-Shields C., Lorenz J., Drossos T., Liu D.C., Skelly C.L. (2013). Median arcuate ligament syndrome in the pediatric population. J. Pediatr. Surg..

[7] Nicolson G.L., Nasralla M., Haier J., Nicolson N.L. (1998). Diagnosis and treatment of Chronic Mycoplasmal Infections in Fibromyalgia and Chronic Fatigue Syndromes: Relation to Gulf War Illness. Biomed. Ther..

[8] Rowe P.C., Underhill R.A., Friedman K.J., Gurwitt A., Medow M.S., Schwartz M.S., Speight N., Stewart J.M., Vallings R., Rowe K.S. Myalgic Encephalomyelitis/Chronic Fatigue Syndrome, Diagnosis and management in young people, A Primer. Front. Paediatr..

[9] Collingridge E.R. (2010). Severe ME/CFS—A Guide to Living.

[10] Rowe K.S. (1997). Double blind randomised control trial to assess the efficacy of intravenous gammaglobulin in the management of Chronic Fatigue Syndrome in adolescents. J. Psychiatr. Res..

